# Trends in Parity and Breast Cancer Incidence in US Women Younger Than 40 Years From 1935 to 2015

**DOI:** 10.1001/jamanetworkopen.2020.0929

**Published:** 2020-03-13

**Authors:** Sarah M. Lima, Rebecca D. Kehm, Katrina Swett, Lou Gonsalves, Mary Beth Terry

**Affiliations:** 1 Mailman School of Public Health, Department of Epidemiology, Columbia University, New York, New York; 2 Connecticut Tumor Registry, Connecticut Department of Public Health, Hartford; 3 Herbert Irving Comprehensive Cancer Center, Columbia University Irving Medical Center, New York, New York

## Abstract

**Importance:**

During the past several decades, breast cancer incidence has been increasing for women younger than 40 years. The increase matches the decrease in parity, an established breast cancer risk factor, but secular trends in incidence have not been examined prior to the 1970s.

**Objective:**

To examine whether secular trends in parity explain the increase in breast cancer incidence among US women aged 25 to 39 years from 1935 to 2015.

**Design, Setting, and Participants:**

This population-based cohort study used population-based aggregate-level data from the Connecticut Tumor Registry (CTR) to examine breast cancer incidence and age-standardized rates among women aged 25 to 39 years from 1935 to 2015. National mean live births were calculated using birth data from the National Vital Statistics System (NVSS) from 1930 to 2015 (allowing for 5-year lag). Linear regression was used to compare a baseline model of year estimating age-adjusted breast cancer incidence rate with a model that adjusted for parity constructs.

**Main Outcomes and Measures:**

Breast cancer incidence rates among women aged 25 to 39 years from 1935 to 2015.

**Results:**

Among women in Connecticut aged 25 to 39 years from 1935 to 2015, incidence of breast cancer for women aged 25 to 39 years increased 0.65% (95% CI, 0.53%-0.77%) per year, from 16.3 breast cancer diagnoses per 100 000 women in 1935 to 38.5 breast cancer diagnoses per 100 000 women in 2015. This increase began nearly 4 decades before the secular decrease in parity (mean [SD] parity peaked at 2.26 [0.87] live births per woman in 1966 and in 2010 had decreased to 1.41 [0.71] live births per woman). Age-specific parity trends explained only 0% to 4% of the variability in incidence over time.

**Conclusion and Relevance:**

These findings suggest that breast cancer incidence for women aged 25 to 39 years has been significantly increasing since the 1930s and cannot be attributed to changes in parity over time.

## Key Points

**Question:**

Can the increase in breast cancer incidence in US women aged 25 to 39 years be explained by trends of decreasing parity?

**Findings:**

In this population-based cohort study including Connecticut women aged 25 to 39 from 1935 to 2015, breast cancer incidence statistically significantly increased by 0.65% per year; after considering parity trends, the annual increase was of similar magnitude and therefore could not explain the trends in breast cancer.

**Meaning:**

These findings suggest that secular trends in parity cannot explain the increasing incidence rate of breast cancer in young women, and this increase cannot be primarily attributed to mammography screening, as the trend analysis shows the increase started prior to screening.

## Teaser

This cohort study examined incidence of breast cancer among US women aged 25 to 39 years from 1935 to 2015 compared with parity data from the same era to determine if the increase in breast cancer incidence could be explained by a decrease in parity.

## Introduction

Breast cancer is the most common malignant tumor among women and has been increasing in incidence for young women in the United States over time.^1^ In a 2019 analysis of Surveillance Epidemiology and End Results (SEER) data from 1975 to 2015,^2^ we observed an annual percentage change (APC) of 0.53% (95% CI, 0.29%-0.78%) per year in breast cancer incidence since 1994 for women younger than 40 years; distant-stage disease increased by 2.73% (95% CI, 2.31%-3.14%) per year since 1975. These APCs cannot be attributed primarily to increased breast cancer screening by mammography, as women are generally screened by mammography after age 40 years,^3,4^ although some guidelines during this period did recommend screening at 35 years and older from 1976 to 1991.^5^ Using data from the oldest tumor registry in the United States, we investigated whether the APC in breast cancer incidence in women younger than 40 years occurred prior to the 1970s and whether secular changes in national mean parity explain this trend.

## Methods

This population-based cohort study used 2 population-based data sources, the Connecticut Tumor Registry (CTR)^6^ and National Vital Statistics System (NVSS).^7,8^ Data are publicly available and deidentified, so this study was exempt from ethical review and informed consent according to Code of Federal Regulations (45 CFR 46.101(b)). This study complies with Strengthening the Reporting of Observational Studies in Epidemiology (STROBE) reporting guideline for cohort studies.

The CTR is the oldest population-based cancer registry in the United States, with data collection starting in 1935. The CTR covers the full state of Connecticut. eTable 1 in the Supplement presents demographic characteristics of Connecticut over time. The registry was more than 75% complete by 1940 to 1944, more than 97% complete in 1968 to 1972, and shortly after, reported to SEER.^9,10^ In this study, we used annual age-adjusted breast cancer incidence rates (based on the 1980 standard population) from 1935 to 2015 for women aged 25 to 39 years. We conducted sensitivity analyses using data from 1940 to 2015 (ie, when CTR data were >75% complete). The NVSS is the oldest intergovernmental database for vital statistics and has included national birth data since 1917. Using distributions of parity in the NVSS historic birth fertility tables, we calculated weighted means of live births (mean parity) from 1930 to 2010. We chose the years 1930 to 2010 to give a 5-year lag for breast cancer incidence data. Additional lag times of 10 and 15 years were included in sensitivity analysis.

### Statistical Analysis

Using linear regression, we compared a baseline model including year as the only estimator of the age-adjusted breast cancer rate, which measured the secular trend in breast cancer incidence, with models adjusted for parity constructs. To fit these models, age-adjusted breast cancer rates were transformed to the log scale. We used the adjusted *R*^2^ to compare variability explained by models unadjusted and adjusted for the parity constructs. We also fit Poisson regression models using crude rates. We ran linear regression models using 5-, 10-, and 15-year lags on additional age groups, including women aged 40 to 54 years, 55 to 69 years, or 70 to 84 years, for a post hoc analysis. We also fit linear regression models for all age groups from 1973 to 2015 as an additional post hoc analysis. We performed a post hoc analysis testing whether trends differed across age groups by including a cross-product term between year and age group in the model. All analyses were performed using SAS statistical software version 9.4 (SAS Institute). A *P* value of .05 for a 2-sided hypothesis test was considered statistically significant.

## Results

**Figure 1.**
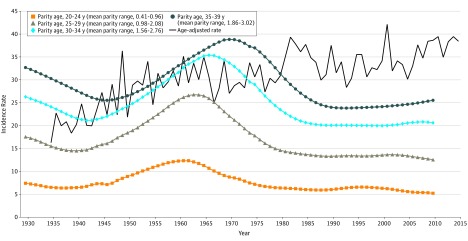
Trends in Age-Adjusted Breast Cancer Incidence for Women Younger Than 40 Years and Mean Parity

Among women in Connecticut aged 25 to 39 years from 1935 to 2015, the APC in breast cancer incidence was 0.65% (95% CI, 0.53%-0.77%) per year, from 16.3 breast cancer diagnoses per 100 000 women in 1935 to 38.5 breast cancer diagnoses per 100 000 women in 2015 (Figure 1). Trends in mean parity were similar among all 4 age groups, with the number of live births peaking during the 1960s baby boom, peaking in 1966 with a mean (SD) parity of 2.26 (0.87) live births per woman. By 1990, parity for all age groups leveled off and remained relatively consistent, with a mean (SD) parity of 1.41 (0.71) live births per woman in 2010. The breast cancer incidence rate continued to increase during periods of increasing, decreasing, and stable parity. Of the total variability in log age-adjusted rate of breast cancer, 57% was explained by year, and adding the parity variables to the base model only explained as much as an additional 4% of the total variability (Figure 1). The APC in breast cancer incidence from 1935 to 2015 was not attenuated after controlling for overall or age-specific mean parity and remained statistically significant (APC, 0.70% [95% CI, 0.56%-0.83%]; *P* < .001) (Table 1). In our post hoc analysis for 1973 to 2015 among young women, the APC per year was 0.40% (95% CI, 0.17%-0.63%) and explained 21% of total variability in log age-adjusted rate of breast cancer; adjusting for parity explained as much as additional 13% of variability.

**Table 1.** Time Trend of Breast Cancer Incidence for Women Younger Than 40 Years

Model	Annual Percentage Change (95% CI)	*P* Value	Adjusted *R*^2^
Time trend	0.65 (0.53-0.77)	<.001	0.57
Adjusted for mean parity by age, y			
All	0.70 (0.56-0.83)	<.001	0.58
20-24	0.73 (0.60-0.85)	<.001	0.61
25-29	0.71 (0.58-0.84)	<.001	0.60
30-34	0.69 (0.56-0.81)	<.001	0.58
35-39	0.67 (0.54-0.80)	<.001	0.57

**Figure 2.**
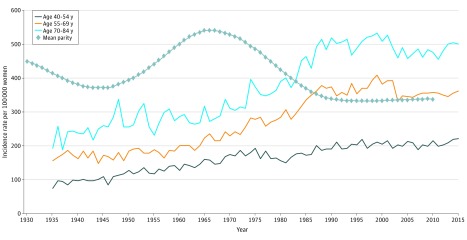
Trends in Age-Adjusted Breast Cancer Incidence for Women Aged 40 Years or Older and Overall Mean Parity The range for mean parity was 1.38 to 2.26 live births per woman.

The APC in breast cancer incidence from 1935 to 2015 was 1.12% (95% CI, 1.02%-1.21%) per year for women aged 40 to 54 years, 1.29% (95% CI, 1.19%-1.39%) per year for women aged 55 to 69 years, and 1.18% (95% CI, 1.08%-1.28%) per year for women aged 70 to 84 years (Figure 2 and Table 2). These APCs were all statistically significantly higher than the APC for women aged 25 to 39 years (*P* < .001); APCs also statistically significantly differed between women aged 40 to 54 years vs 55 to 69 years (*P* = .02). After adjusting for mean parity, APCs in breast cancer incidence for these age groups were not markedly changed and remained statistically significant (age 40-54 years: APC, 0.61% [95% CI, 0.46%-0.77%]; age 55-69 years: APC, 0.70% [95% CI, 0.47%-0.92%]; age 70-84 years: APC, 0.78% [95% CI, 0.54%-1.03%]; *P* < .001). When we narrowed the period to 1973 to 2015 to allow for comparison with prior studies using SEER data, the age-adjusted cancer incidence rates for women 40 years and older were attenuated and no longer statistically significant after adjusting for mean parity. For example, the unadjusted APC of 0.61% (95% CI, 0.46%-0.77%; *P* < .001) per year for women aged 40 to 54 years from 1973 to 2015 was reduced to 0.24% (95% CI, –0.07% to 0.55%; *P* = .13) per year after adjusting for mean parity. In the sensitivity analysis of incidence data from 1940 to 2015 among women 40 years and older vs younger than 40 years, our results did not change. Using additional lag times of 10 and 15 years did not alter our conclusions (eTable 2 and eTable 3 in the Supplement). Using Poisson regression models also did not change our conclusions (eTable 4 in the Supplement).

**Table 2.** Time Trend of Breast Cancer Incidence for Women Aged 40 Years or Older

Age, y	Time Trend			Time Trend Adjusted for Mean Parity		
	APC (95% CI)	*P* Value	Adjusted *R*^2^	APC (95% CI)	*P* Value	Adjusted *R*^2^
1935-2015						
40-54	1.12 (1.02 to 1.21)	<.001	0.87	1.22 (1.13 to 1.31)	<.001	0.91
55-69	1.29 (1.19 to 1.39)	<.001	0.89	1.30 (1.19 to 1.42)	<.001	0.89
70-84	1.18 (1.08 to 1.28)	<.001	0.86	1.13 (1.02 to 1.24)	<.001	0.87
1973-2015						
40-54	0.61 (0.46 to 0.77)	<.001	0.59	0.24 (–0.07 to 0.55)	.13	0.64
55-69	0.70 (0.47 to 0.92)	<.001	0.47	–0.47 (–0.72 to –0.22)	<.001	0.85
70-84	0.78 (0.54 to 1.03)	<.001	0.49	–0.47 (–0.75 to –0.18)	.003	0.84

Abbreviation: APC, annual percentage change.

## Discussion

This population-based cohort study found that with every 1-year increase from 1935 to 2015, the incidence of breast cancer for women aged 25 to 39 years increased by approximately 0.65%. The absolute incidence rate of breast cancer changed from 16.3 breast cancer diagnoses per 100 000 women in 1935 to 38.5 breast cancer diagnoses per 100 000 in 2015, which translates to a relative rate of 2.4. This result is important because it establishes upward trends in breast cancer incidence prior to the 1970s. We also found that the increase in breast cancer incidence began more than 3 decades before the secular decrease in parity; thus, the increase in breast cancer cannot be attributed to declining trends in parity. Because our age of interest is younger than 40 years, the significant increase in breast cancer incidence also cannot be explained by increased mammography starting in the 1970s. The trend analysis also shows that the increase in breast cancer began long before routine mammography was initiated. Past studies investigating the association of screening with breast cancer incidence have corroborated this finding, concluding that screening accounts for little of the long-term incidence increase, particularly among women younger than 40 years.^11^

### Study Context

A 2018 study by Pfeiffer et al^12^ found an association of decreasing parity with increasing breast cancer incidence trends, but their study focused on women diagnosed with breast cancer from 1980 to 2011. Because most studies on breast cancer incidence use data from the 1970s and onward, major changes in parity, such as the increase during the baby boom, are not fully captured. We demonstrated this by also restricting our analyses to the 1970s and onward, during which period we found the APC was dramatically reduced, supporting the inference that more recent declines in parity may explain the increase in cancer incidence, which is negated with an extended time period. When we conducted post hoc analyses just using data from the 1970s forward, parity did explain some of the increase. Although recent parity trends track with breast cancer incidence trends and can partially explain the increased incidence, they do not explain most of the increase, and the historical trends using a much longer time lens provide a strong argument against parity being the primary factor associated with increasing breast cancer incidence, particularly in younger women.

### Limitations and Strengths

This study has some limitations. Although we had population-based trend data for cancer and parity information, we were not able to link the 2 sources of data; thus, this study may be confounded by factors associated with the trends in both. However, we did focus on the most consistent breast cancer risk factor—parity—in examining these trends. Although we only had access to nationwide data for parity trends data, Connecticut has shown similar parity trends to the national mean.^13^ Connecticut has one of the highest breast cancer incidence rates within the United States.^14,15^ While this may reduce the generalizability of our study, we believe the importance of using data available prior to the baby boom outweighs concerns of external validity. Generalizability may also be limited by the racial composition of Connecticut. We were unable to include data on other risk factors, such as body mass index, mammographic screening rates, or age at first birth, as most of these data are not available until more recent decades after the baby boom.

Our study also has some strengths. Key strengths of the study include the length of time studied starting before the baby boom and the use of a population-based registry for cancer data and survey information for parity data.

## Conclusions

This study found that breast cancer incidence has been significantly increasing for the past 80 years, with the increase beginning at least a decade before the baby boom. Findings from our time-series analysis suggest that this increase cannot be explained primarily by secular trends in parity.

## Notes

Supplement. **eTable 1.** Distribution of Racial Groups in Connecticut Over Time **eTable 2.** Results for Linear Regression Models by Age Group Using a 10-year Lag **eTable 3.** Results for Linear Regression Models by Age Group Using a 15-year Lag **eTable 4.** Incidence Rate Ratio for Every 10-year Increase for Women Aged 25 to 39 Years
