# Supplementary material for: Trends in Parity and Breast Cancer Incidence in US Women Younger Than 40 Years From 1935 to 2015
Source: JAMA Netw Open. 2020 Mar 13;3(3):e200929. doi: 10.1001/jamanetworkopen.2020.0929 (PMC7070232; doi:10.1001/jamanetworkopen.2020.0929)
Supplement: Supplement. — eTable 1. Distribution of Racial Groups in Connecticut Over Time eTable 2. Results for Linear Regression Models by Age Group Using a 10-year Lag eTable 3. Results for Linear Regression Models by Age Group Using a 15-year Lag eTable 4. Incidence Rate Ratio for Every 10-year Increase for Women Aged 25 to 39 Years [file jamanetwopen-3-e200929-s001.pdf]

Supplementary Online Content

Lima SM, Kehm RD, Swett K, Gonsalves L, Terry MB. Trends in parity and breast cancer incidence in US women younger than 40 years from 1935 to 2015. *JAMA Netw Open*. 2020;3(3):e200929. doi:10.1001/jamanetworkopen.2020.0929

eTable 1. Distribution of Racial Groups in Connecticut Over Time
eTable 2. Results for Linear Regression Models by Age Group Using a 10-year Lag
eTable 3. Results for Linear Regression Models by Age Group Using a 15-year Lag
eTable 4. Incidence Rate Ratio for Every 10-year Increase for Women Aged 25 to 39 Years

This supplementary material has been provided by the authors to give readers additional information about their work.

© 2020 Lima SM et al. *JAMA Network Open.*

eTable 1. Distribution of Racial Groups in Connecticut Over Time

Year	White	Black	American Indian, Eskimo, and Aleut	Asian and Pacific Islander	Other race
2015	74%	13%	1%	5%	5%
2010	78%	10%	0%	4%	6%
2000	82%	9%	0%	2%	4%
1990	87%	8%	0%	2%	3%
1980	90%	7%	0%	1%	2%
1970	94%	6%	0%	0%	0%
1960	96%	4%	0%	0%	0%
1950	97%	3%	0%	0%	0%
1940	98%	2%	0%	0%	0%
1930	98%	2%	0%	0%	0%
1920	98%	2%	0%	0%	0%

Sources: “Population of Connecticut:Census 2010 and 2000 Interactive Map, Demographics, Statistics, Quick Facts.” CensusViewer, censusviewer.com/state/CT; Gibson, Campbell, and Kay Jung. Historical census statistics on population totals by race, 1790 to 1990, and by Hispanic origin, 1790 to 1990, for the United States, regions, divisions, and states. Washington, DC: US Census Bureau, 2002.

© 2020 Lima SM et al. *JAMA Network Open.*

eTable 2. Results for Linear Regression Models by Age Group Using a 10-year Lag

Age group	Model	APC (CI)	ADJ R SQ	P
**25-39**	Time trend	0.65 (0.53, 0.77)	0.570	<0.0001
	Time trend adjusted for average parity of all ages	0.66 (0.53, 0.79)	0.568	<0.0001
	Time trend adjusted for average parity of 20-24 olds	0.68 (0.55, 0.8)	0.579	<0.0001
	Time trend adjusted for average parity of 25-29 olds	0.68 (0.55, 0.81)	0.580	<0.0001
	Time trend adjusted for average parity of 30-34 olds	0.65 (0.51, 0.78)	0.567	<0.0001
	Time trend adjusted for average parity of 35-39 olds	0.66 (0.53, 0.79)	0.568	<0.0001
**40-54**	Time trend adjusted for average parity of all ages	1.18 (1.08, 1.27)	0.8849	<0.0001
	Time trend adjusted for average parity of 20-24 olds	1.19 (1.11, 1.27)	0.91	<0.0001
	Time trend adjusted for average parity of 25-29 olds	1.19 (1.11, 1.27)	0.9102	<0.0001
	Time trend adjusted for average parity of 30-34 olds	1.13 (1.03, 1.23)	0.8722	<0.0001
	Time trend adjusted for average parity of 35-39 olds	1.15 (1.06, 1.25)	0.8788	<0.0001
	Time trend adjusted for average parity of 40-44 olds	1.14 (1.04, 1.24)	0.8735	<0.0001
**55-69**	Time trend adjusted for average parity of all ages	1.37 (1.27, 1.47)	0.9072	<0.0001
	Time trend adjusted for average parity of 20-24 olds	1.28 (1.18, 1.39)	0.8867	<0.0001
	Time trend adjusted for average parity of 25-29 olds	1.3 (1.2, 1.41)	0.8871	<0.0001
	Time trend adjusted for average parity of 30-34 olds	1.34 (1.24, 1.44)	0.8967	<0.0001
	Time trend adjusted for average parity of 35-39 olds	1.37 (1.27, 1.46)	0.9126	<0.0001
	Time trend adjusted for average parity of 40-44 olds	1.41 (1.34, 1.49)	0.9423	<0.0001
**70-84**	Time trend adjusted for average parity of all ages	1.2 (1.09, 1.31)	0.8617	<0.0001
	Time trend adjusted for average parity of 20-24 olds	1.13 (1.03, 1.24)	0.876	<0.0001
	Time trend adjusted for average parity of 25-29 olds	1.15 (1.04, 1.26)	0.8665	<0.0001
	Time trend adjusted for average parity of 30-34 olds	1.18 (1.07, 1.29)	0.8603	<0.0001
	Time trend adjusted for average parity of 35-39 olds	1.21 (1.11, 1.32)	0.8663	<0.0001
	Time trend adjusted for average parity of 40-44 olds	1.28 (1.18, 1.37)	0.8984	<0.0001

© 2020 Lima SM et al. *JAMA Network Open.*

eTable 3. Results for Linear Regression Models by Age Group Using a 15-year Lag

Age group	Model	APC (CI)	ADJ R SQ	P
25-39	Time trend	0.65 (0.53, 0.77)	0.570	<0.0001
	Time trend adjusted for average parity of all ages	0.64 (0.51, 0.77)	0.568	<0.0001
	Time trend adjusted for average parity of 20-24 olds	0.66 (0.53, 0.78)	0.571	<0.0001
	Time trend adjusted for average parity of 25-29 olds	0.66 (0.54, 0.79)	0.573	<0.0001
	Time trend adjusted for average parity of 30-34 olds	0.63 (0.51, 0.76)	0.571	<0.0001
	Time trend adjusted for average parity of 35-39 olds	0.64 (0.52, 0.77)	0.568	<0.0001
40-54	Time trend adjusted for average parity of all ages	1.15 (1.05, 1.25)	0.8755	<0.0001
	Time trend adjusted for average parity of 20-24 olds	1.15 (1.07, 1.24)	0.8949	<0.0001
	Time trend adjusted for average parity of 25-29 olds	1.15 (1.06, 1.24)	0.8877	<0.0001
	Time trend adjusted for average parity of 30-34 olds	1.13 (1.03, 1.22)	0.8712	<0.0001
	Time trend adjusted for average parity of 35-39 olds	1.13 (1.04, 1.23)	0.8731	<0.0001
	Time trend adjusted for average parity of 40-44 olds	1.12 (1.02, 1.22)	0.8706	<0.0001
55-69	Time trend adjusted for average parity of all ages	1.4 (1.31, 1.48)	0.9345	<0.0001
	Time trend adjusted for average parity of 20-24 olds	1.31 (1.21, 1.41)	0.8912	<0.0001
	Time trend adjusted for average parity of 25-29 olds	1.33 (1.23, 1.43)	0.9013	<0.0001
	Time trend adjusted for average parity of 30-34 olds	1.35 (1.25, 1.44)	0.9082	<0.0001
	Time trend adjusted for average parity of 35-39 olds	1.39 (1.31, 1.46)	0.944	<0.0001
	Time trend adjusted for average parity of 40-44 olds	1.41 (1.34, 1.48)	0.949	<0.0001
70-84	Time trend adjusted for average parity of all ages	1.24 (1.14, 1.35)	0.88	<0.0001
	Time trend adjusted for average parity of 20-24 olds	1.17 (1.07, 1.28)	0.8615	<0.0001
	Time trend adjusted for average parity of 25-29 olds	1.19 (1.08, 1.3)	0.8611	<0.0001
	Time trend adjusted for average parity of 30-34 olds	1.2 (1.09, 1.3)	0.8621	<0.0001
	Time trend adjusted for average parity of 35-39 olds	1.26 (1.17, 1.35)	0.9042	<0.0001
	Time trend adjusted for average parity of 40-44 olds	1.3 (1.22, 1.38)	0.9302	<0.0001

© 2020 Lima SM et al. *JAMA Network Open.*

eTable 4. Incidence Rate Ratio for Every 10-year Increase for Women Aged 25 to 39 Years

**Model covariates**	**IRR (95% CI)**	**p-value**
Year	1.068(1.055, 1.082)	<0.0001
Year + Average Parity	1.070(1.055, 1.084)	<0.0001
Year + Parity among 20-24	1.078(1.062, 1.094)	<0.0001
Year + Parity among 25-29	1.071(1.055, 1.090)	<0.0001
Year + Parity among 30-34	1.067(1.051, 1.082)	<0.0001
Year + Parity among 35-39	1.064(1.049, 1.079)	<0.0001

© 2020 Lima SM et al. *JAMA Network Open.*
